# Syndrome de Poland

**DOI:** 10.11604/pamj.2015.22.124.7972

**Published:** 2015-10-12

**Authors:** Manel Jellouli, Tahar Gargah

**Affiliations:** 1 Service de Pédiatrie, Hôpital Charles Nicolle, Tunis, Tunisie

**Keywords:** Syndrome de poland, enfant, malformations, poland syndrome, infant, malformations

## Images in medicine

Le syndrome de Poland correspond à l'absence ou l'hypoplasie unilatérale du grand pectoral associée à des anomalies homolatérales de la main, le plus souvent une symbrachydactylie. Garçon âgé de 5 ans, issu d'un mariage non consanguin et d'une grossesse de déroulement normal sans notion de prise médicamenteuse par la mère. A la naissance, la malformation au niveau de la main gauche a été décelée. A l'examen physique, l'enfantétait eutrophique,le quotient intellectuel était normal pour l’âge. L'examen cardiaque abdominal et neurologique était sans anomalies. Les organes génitaux externes étaientde type masculin bien différenciés. Il présentait deux anomalies malformatives cliniquement visibles: une brachymésophalangie gauche(A) et une asymétrie thoracique avec hypoplasie du grand pectoral gauche avec absence du chef long (B). La fonction rénale était normale, la numération de la formule sanguine était normale de même que le bilan d'hémostase. Le caryotype sanguin trouvait la formule 46XY. L’échographie abdomino-rénale était sans anomalies, l’échographie cardiaque montrait une fonction VG normale sans atteinte des autres cavités et sans valvulopathie. L'examen ophtalmologique était normal. Il s'agit d'un syndrome de Poland avec hypoplasie du grand pectoral et une brachymésophalangie gauche sans malformations associées en particulier pas d'atteinte cardiaque ou d'anomalie génito-urinaire. Le patient a été confié aux orthopédistes pour appareillage prothétique.

**Figure 1:**
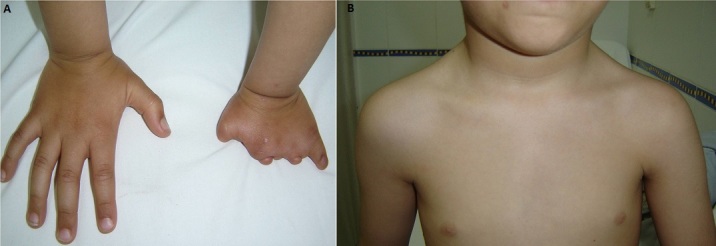
(A) brachymésophalangie gauche; (B) hypoplasie du grand pectoral gauche

